# MRSA in pig farms: a prospective cohort study

**DOI:** 10.1186/1753-6561-5-S6-P19

**Published:** 2011-06-29

**Authors:** BV Cleef, BV Benthem, R Teuwen, J Kluytmans

**Affiliations:** 1 Department of Medical Microbiology and Infection Prevention, VU University Medical Centre, Amsterdam, Netherlands; 2 Centre for Infectious Disease Control Netherlands, RIVM National Institute for Public Health and the Environment, Bilthoven, Netherlands; 3 Laboratory for Microbiology and Infection Control, Amphia Hospital, Breda, Netherlands

## Introduction / objectives

The aims of this study were to determine the prevalence, determinants and dynamics of human carriage of livestock associated methicillin-resistant *Staphylococcus aureus* (MRSA) in pig farms.

## Methods

In this prospective cohort study in 50 pig farms in The Netherlands, human nasal samples were collected on 5 time points in 8 months. At start of the study, throat samples and environmental samples were taken. Persistent carriers were defined as persons with 100% of nasal samples positive for MRSA, non-carriers had no MRSA and intermittent carriers had at least one positive sample. Data collection is still ongoing (total follow-up 1 year).

## Results

In total, 281 persons participated in the study. At start of the study, 21% of household members (31/147) and 68% of farmers/employees (90/132) were MRSA positive in nose or throat. Fifty-four persons were persistent MRSA carriers (54/281=19%): 5 household members (5/149=4%) and 49 farmers/employees (49/132=37%). Furthermore, 79 intermittent carriers and 148 non-carriers were found. Sixty-seven percent (33/49) of the residences and 80% (40/50) of the stables harboured MRSA. Working in the stables on a daily base and presence of MRSA in throat samples were significantly associated with persistent carriage (OR=12.7, 95%CI 4.1-39.3, and OR=5.0, 95%CI 2.3-10.9, respectively).

## Conclusion

Persistent MRSA nasal carriage is common in persons working at pig farms. A possible explanation is the frequent exposure to high amounts of MRSA in the stables. Working in the stables still is an important factor in persistent carriage, as is throat carriage.

## Disclosure of interest

None declared.

